# Orthodontic creative mechanics

**DOI:** 10.1590/2177-6709.31.1.e26spe1

**Published:** 2026-04-17

**Authors:** Andre Wilson MACHADO

**Affiliations:** 1 Federal University of Bahia, Adjunct Professor, Department of Orthodontics (Salvador/BA, Brazil).

**Keywords:** Orthodontics, Creativity, Mechanics, Tooth movement techniques, Orthodontic appliance design, Ortodontia, Criatividade, Mecânica, Técnicas de movimentação dentária, Desenho de aparelho ortodôntico

## Abstract

**Introduction:**

In-depth knowledge of orthodontic mechanics is a mandatory aspect for all orthodontists, regardless of the technique, material, or appliance used. Following this reasoning, in addition to notorious knowledge, the clinician needs to be creative to deal with the countless possibilities inherent to clinical diversity.

**Objective:**

The objective of this article is to present 10 clinical situations in which the orthodontist’s inventive capacity is a fundamental element in selecting ideal mechanics to solve different clinical challenges.

**Results:**

Incorporating creativity enabled efficient resolution of all clinical situations.

**Conclusions:**

Stimulating creativity is essential to prepare professionals capable of innovating responsibly.

## RESUMO

**Introdução:**

O profundo conhecimento em mecânica ortodôntica é aspecto mandatório para todos os ortodontistas, independentemente da técnica, material ou aparelho utilizado. Seguindo esse raciocínio, além de conhecimento técnico, o clínico precisa ter criatividade para lidar com as inúmeras possibilidades inerentes à diversidade clínica.

**Objetivo:**

O objetivo deste artigo é apresentar dez situações clínicas nas quais a capacidade inventiva do ortodontista foi elemento fundamental na seleção da mecânica ideal para a resolução de diferentes desafios clínicos.

**Resultados:**

Com a incorporação da criatividade, foi possível resolver todas as dez situações clínicas com bastante eficiência.

**Conclusões:**

Estimular a criatividade desde a formação acadêmica é fundamental para preparar profissionais capazes de inovar com responsabilidade.

## INTRODUCTION

For Orthodontics to be practiced with excellence, the clinician must possess an extensive set of both theoretical and practical knowledge. Among the competencies that fulfill this dual qualification, orthodontic mechanics undoubtedly holds an essential position.^1^

At the same time, especially for beginners in the orthodontic career, numerous questions often arise^2^: Which orthodontic technique should I use? Should I work with brackets or aligners? If brackets are chosen, which prescription is most suitable? Should I use conventional or self-ligating brackets? And if aligners are used, which system is preferable?

Although there are no prompt answers to these questions, mastering orthodontic mechanics makes these decisions more straightforward.^1^ ^-^ ^3^ However, even with this knowledge, clinicians must employ creativity to address the many possibilities arising from clinical diversity.^4^ ^-^ ^7^

In clinical contexts, creativity is often necessary to modify or adapt conventional orthodontic mechanics. Common examples include using orthodontic buttons, lingual holding arches, palatal bars, and/or mini-implants in unconventional ways to enable complex tooth movements while minimizing side effects.^4^ ^,^ ^5^ ^,^ ^7^ ^,^ ^8^ The choice of application sites, mechanical design, and the direction of applied forces in each clinical situation requires innovative thinking and refined clinical reasoning.

Thus, the objective of this article is to present ten clinical situations in which the orthodontist’s creativity was a decisive factor in selecting the optimal mechanics for resolving various clinical scenarios.

## ORTHODONTIC CREATIVE MECHANICS

### CLINICAL SITUATION 1

#### Correction of maxillary premolars rotation with a palatal bar

This example illustrates the relevance of creativity in orthodontic practice, as the correction of premolar rotation through conventional mechanics remains a challenging procedure, regardless of whether brackets or aligners are employed. Notably, when aligners are used, the efficacy of such correction is further compromised due to the inherently low predictability of this specific type of tooth movement.^9^

**Figure 1:**
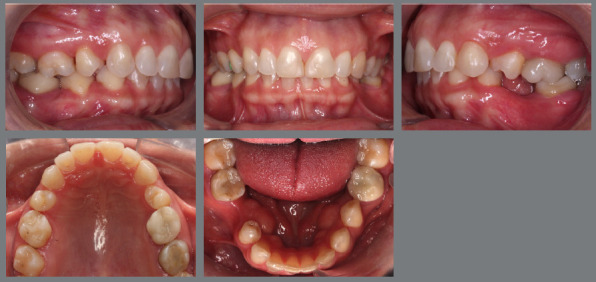
Initial intraoral photographs.

**Figure 2:**
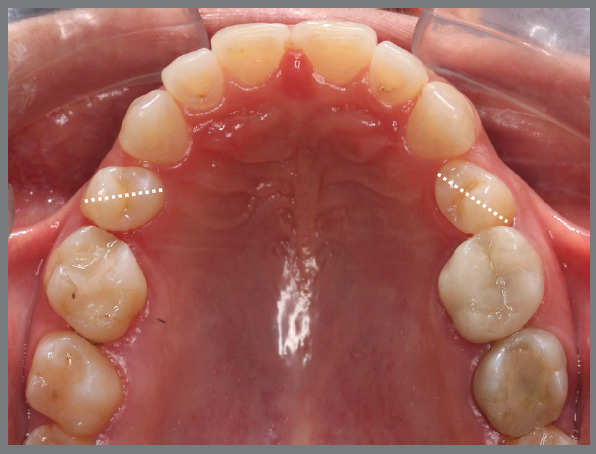
Maxillary occlusal intraoral photograph illustrating the severity of premolar rotations.

The present patient wished to undergo orthodontic treatment with aligners. She presented with a Class II, division 1, malocclusion, deep bite, and multiple diastemas (Fig. 1). Clinically, the most concerning feature was the rotation of the maxillary second premolars (Fig. 2). Her main complaint was related to smile esthetics and a desire to rehabilitate edentulous spaces.

Initially, the decision was made to correct the maxillary premolar rotations before starting treatment with aligners.

**Figure 3:**
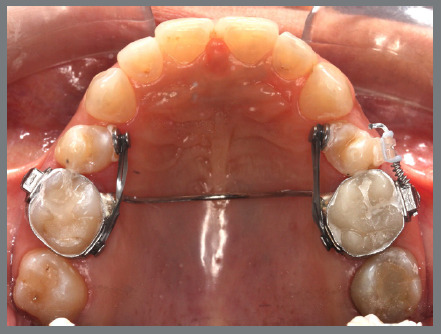
Maxillary occlusal intraoral photograph of the mechanics applied for the correction of rotations.

**Figure 4:**
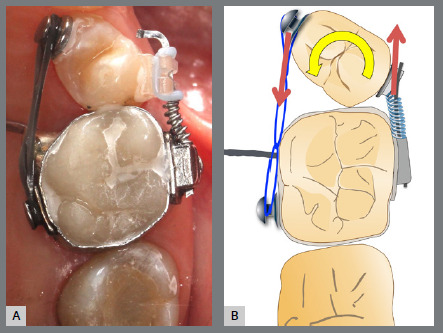
Close-up photograph **(A)** and schematic illustration **(B)** of the force and moment system applied in the mechanics.

**Figure 5:**
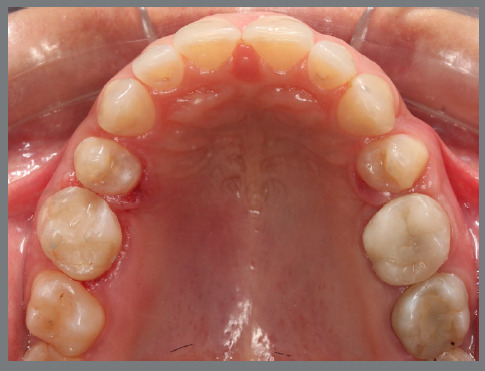
Maxillary occlusal intraoral photograph illustrating the outcome of the mechanics applied.

**Figure 6:**
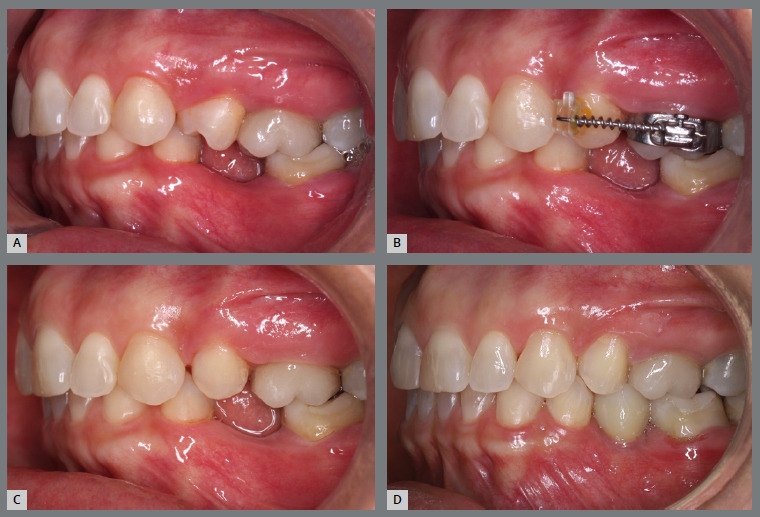
Left-side intraoral photographs illustrating the main stages of clinical case progression: **A)** initial; **B)** after correction of premolar rotation; **C)** initial stage of treatment with aligners; and **D)** post-treatment result.

**Figure 7:**
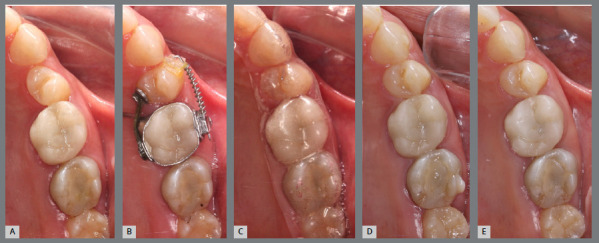
Maxillary occlusal intraoral photographs illustrating the main stages of clinical case progression: **A)** initial; **B)** after correction of premolar rotation; **C)** retention phase following rotation correction and waiting period before aligner therapy; **D)** aligner treatment phase; and **E)** post-treatment result.

A rigid (0.9 mm) palatal bar was used, anchored to the maxillary first molars. On the rotated premolars, brackets were bonded to the buccal surfaces and buttons, to the palatal surfaces. To create a couple of forces, an elastic chain was applied palatally (distal force) and an open-coil spring was placed buccally (mesial force) (Figs. 3 and 4). After three months, the premolars’ positions were corrected, and an acetate retainer was placed to maintain the result while awaiting the arrival of the aligners (Fig. 5). Figures 6 and 7 illustrate the main events in the case evolution.

### CLINICAL SITUATION 2

#### Correction of a maxillary premolar rotation with a modified Nance appliance

Similar to the previous case, creativity was integrated into the treatment plan to correct a severe rotation of a maxillary right second premolar before initiating conventional orthodontic treatment with brackets.

**Figure 8:**
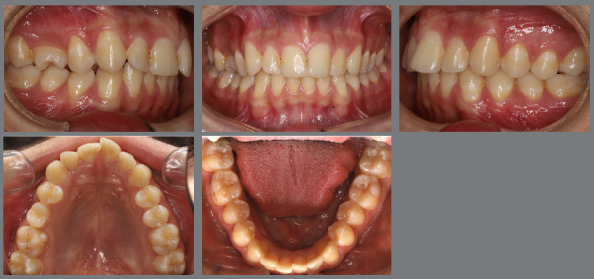
Initial intraoral photographs.

**Figure 9:**
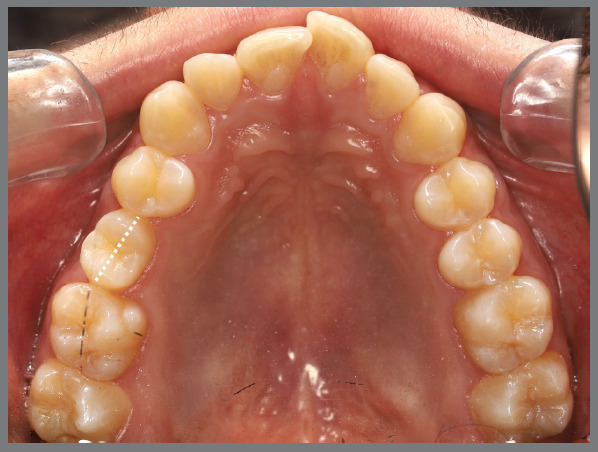
Maxillary occlusal intraoral photograph illustrating the severity of premolar rotation.

The present patient presented with a Class II, division 1, malocclusion, with mild crowding and severe rotation of the maxillary right second premolar. His main complaint was improving smile esthetics (Figs. 8 and 9).

**Figure 10:**
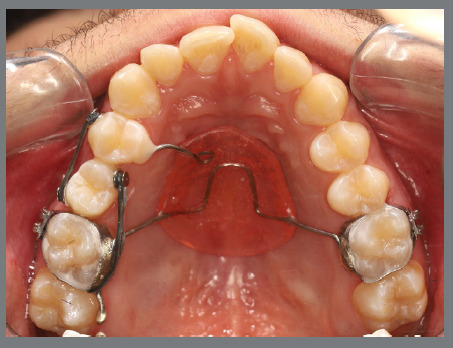
Maxillary occlusal intraoral photograph of the mechanics applied for rotation correction.

**Figure 11:**
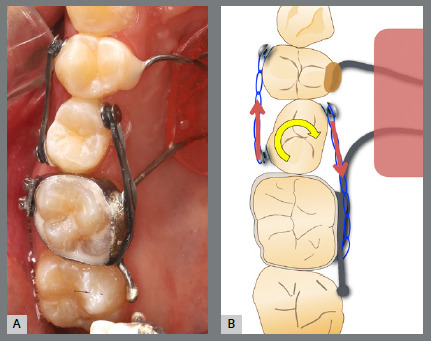
Close-up photograph **(A)** and schematic illustration **(B)** of the force and moment system applied in the mechanics.

Before bonding maxillary brackets, a modified Nance appliance was fabricated, anchored to the maxillary first molars. Inside the acrylic button, a wire extension was built to reach the palatal surface of the maxillary right first premolar. Two buttons were bonded to the right second premolar (palatal and buccal surfaces) to generate a couple of forces for derotation. To avoid undesirable movement of the first premolar, it was bonded to the prefabricated wire extension (Figs. 10 and 11).

**Figure 12:**
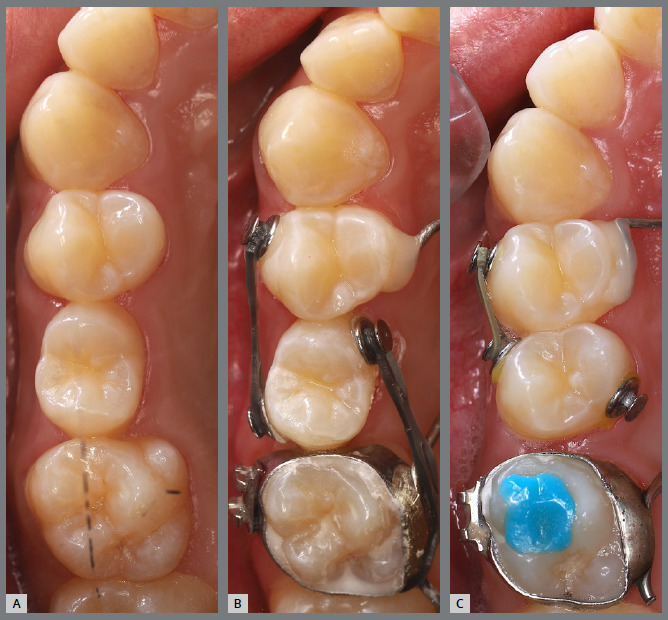
Maxillary occlusal intraoral photographs illustrating the main stages of clinical case progression: **A)**initial; **B)**mechanics employed; and **C)**post-rotation correction result.

**Figure 13:**
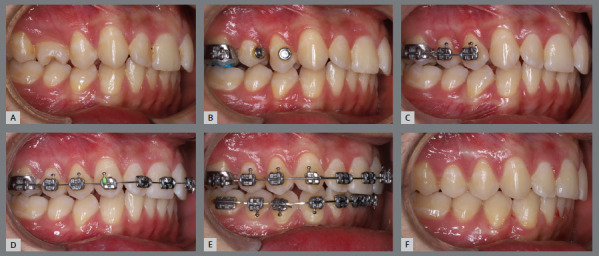
Right-side intraoral photographs illustrating the main stages of clinical case progression: **A)** initial; **B)** after correction of premolar rotation; **C)** bracket bonding on premolars; **D)** placement of the maxillary fixed appliance and initiation of alignment and leveling; **E)** placement of the full orthodontic appliance and completion of alignment and leveling phase; and **F)** final result.

After two months without complete correction, occlusal bite turbos were placed on the maxillary first molars. Three months later, full correction was achieved. At that point, the buttons were removed, brackets were bonded to the premolars, and a 0.017 x 0.025-in nickel-titanium segmented archwire was inserted. Full maxillary brackets were then placed to initiate alignment and leveling, followed by correction of the sagittal discrepancy using Class II elastics. Figures 12 and 13 illustrate the main events in the case evolution.

### CLINICAL SITUATION 3

#### Correction of mandibular premolar rotations using a lingual holding arch

As in the previous cases, creativity was employed to correct the rotations of all four mandibular premolars before initiating treatment with aligners.

**Figure 14:**
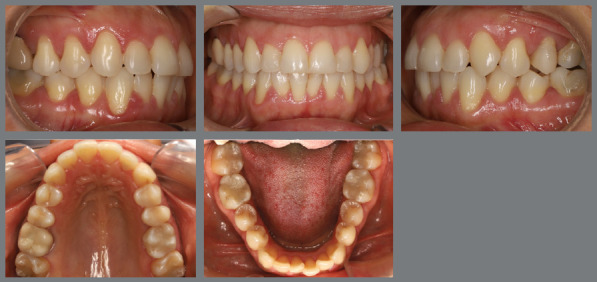
Initial intraoral photographs.

**Figure 15:**
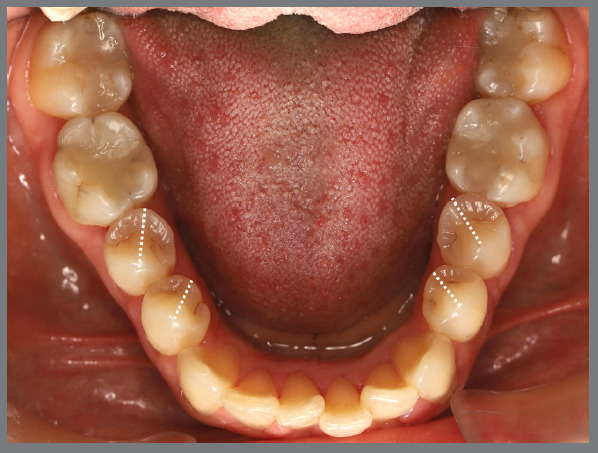
Mandibular occlusal intraoral photograph illustrating the severity of premolar rotations.

The patient presented with a Class I malocclusion, dental crowding, and moderate rotation of the mandibular premolars (Figs. 14 and 15). Her chief complaint was to improve smile esthetics, and she preferred treatment with aligners.

**Figure 16:**
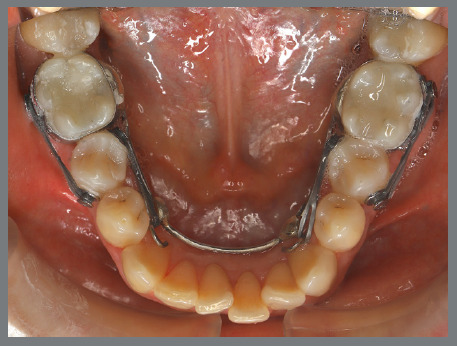
Mandibular occlusal intraoral photograph of the mechanics applied for rotation correction.

**Figure 17:**
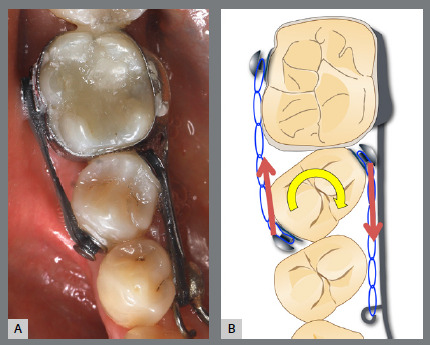
Close-up photograph **(A)** and schematic illustration **(B)** of the force and moment system applied in the mechanics.

Before starting aligner therapy, a rigid mandibular lingual holding arch was anchored to the first molars, with buttons welded to the buccal surfaces of the molar bands and two hooks welded to the internal arch near the canine regions. Initially, buttons were bonded to the mandibular second premolars (lingual and buccal surfaces) to create a couple of forces for derotation. The palatal elastic chain was attached to the soldered hook (mesial force), while the buccal elastic chain was attached to the buccal button on the molar band (distal force) (Figs. 16 and 17).

**Figure 18:**
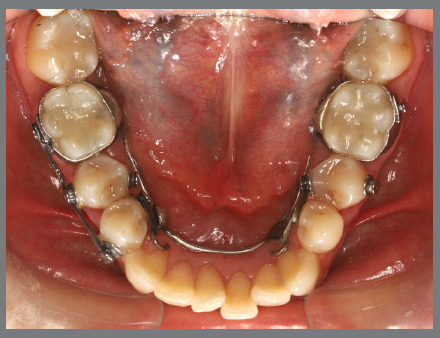
Mandibular occlusal intraoral photograph illustrating the outcome of the mechanics employed for correction of second premolar rotations.

**Figure 19:**
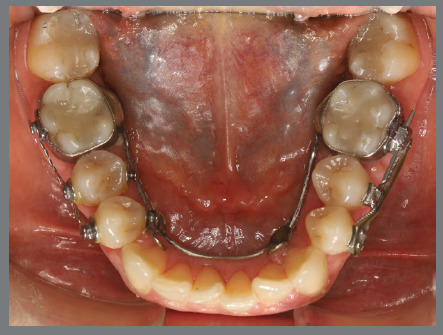
Mandibular occlusal intraoral photograph illustrating the outcome of the mechanics employed for correction of first premolar rotations.

**Figure 20:**
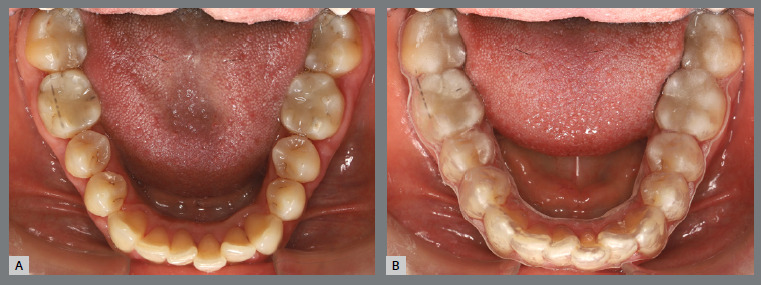
Mandibular occlusal intraoral photographs illustrating: **A)** outcome of the mechanics employed; and **B)** use of an acetate retainer during the waiting period before aligner therapy.

**Figure 21:**
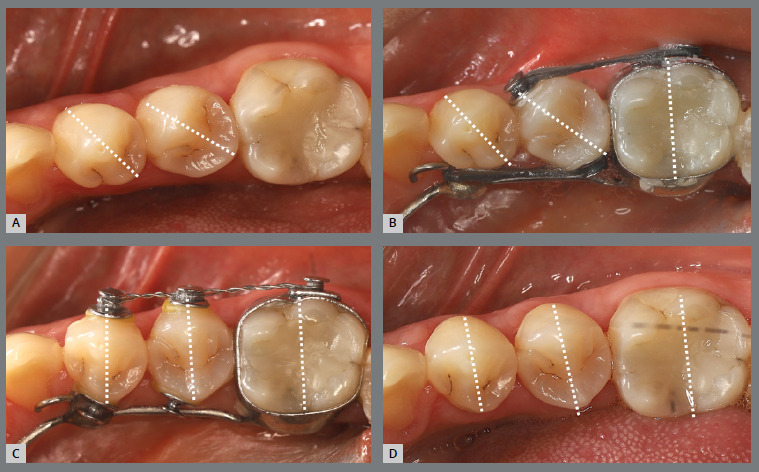
Close-up mandibular intraoral photographs illustrating the main stages of clinical case progression: **A)** initial; **B)** mechanics for second premolar rotation; **C)** outcome after correction of all premolar rotations; and **D)** post-mechanics outcome and waiting period before aligner therapy.

After three months, rotation correction of the mandibular second premolars was clinically achieved, with overcorrection on the left side (Fig. 18). Buttons were then bonded to the first premolars, and a similar mechanism was applied to correct their rotations (Fig. 19). After two additional months, all four premolars were corrected, the appliances were removed, and an acetate retainer was placed to maintain the result until the aligners arrived (Fig. 20). Figure 21 illustrate the main events in the case evolution. 

### CLINICAL SITUATION 4

#### Correction of mandibular canine rotations using a lingual holding arch

**Figure 22:**
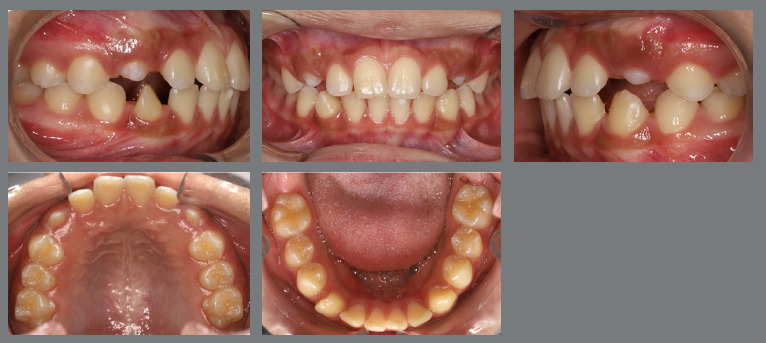
Initial intraoral photographs.

**Figure 23:**
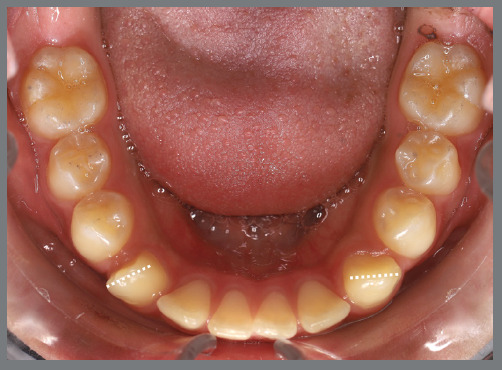
Mandibular occlusal intraoral photograph illustrating the severity of canine rotations.

The patient, aged 12, presented with a Class I malocclusion and significant rotation of the mandibular canines (Figs. 22 and 23). Before bonding the fixed appliance, creativity was employed to correct the canine rotations.

**Figure 24:**
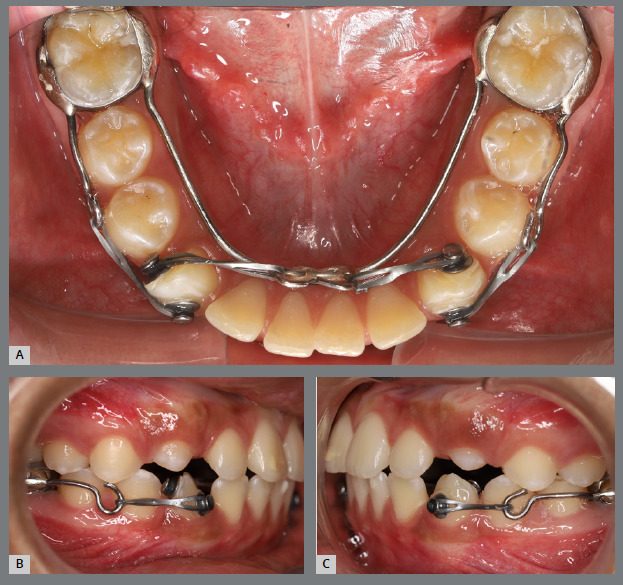
Intraoral photographs illustrating the mechanics employed for rotation correction: **A)**mandibular occlusal view; **B)**right lateral view; and **C)**left lateral view.

**Figure 25:**
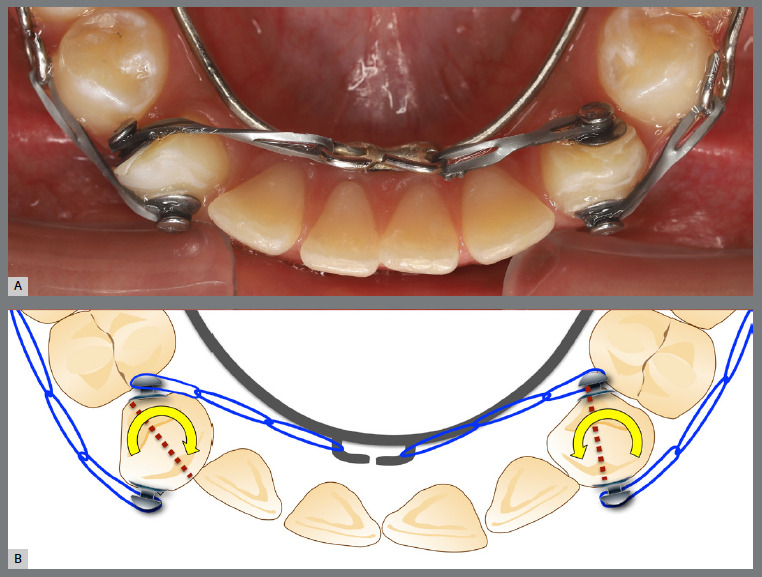
Close-up photograph **(A)** and schematic illustration **(B)** of the mechanics employed.

A rigid (0.9 mm) mandibular lingual arch was placed, positioned away from the incisors, and anchored to the first molars. This device had two notable features: (a) buccal hook extensions on both sides reaching the first premolar region, to serve as anchorage points, and (b) hooks soldered to the lingual arch at the lingual surfaces of the central incisors, also serving as anchorage points. Two buttons were bonded to each canine (buccal and lingual surfaces) to create couples of forces for derotation (Figs. 24 and 25).

**Figure 26:**
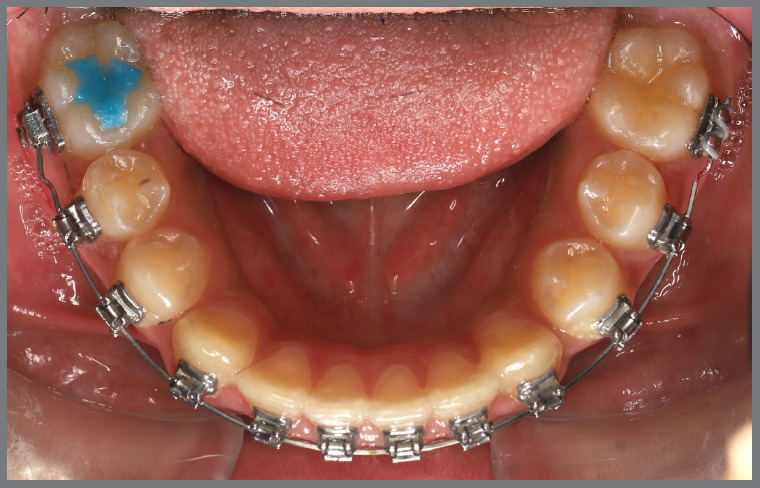
Mandibular occlusal intraoral photograph illustrating the outcome of the mechanics employed.

**Figure 27:**
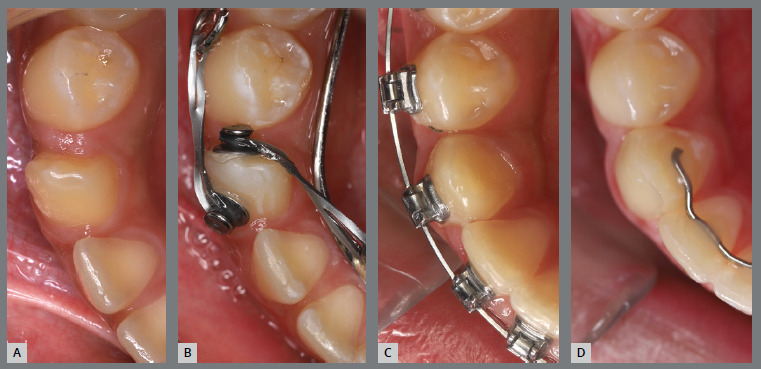
Mandibular occlusal intraoral photographs illustrating the main stages of clinical case progression: **A)** initial; **B)** after correction of canine rotation; **C)** outcome of the mechanics; and **D)** post-treatment result.

After four months, the rotations were corrected, the fixed appliance was bonded, and alignment and leveling were initiated (Fig. 26). Figure 27 illustrates the main events in the case evolution.

### CLINICAL SITUATION 5

#### Intrusion and buccal tipping of a mandibular second molar using a lingual holding arch

**Figure 28:**
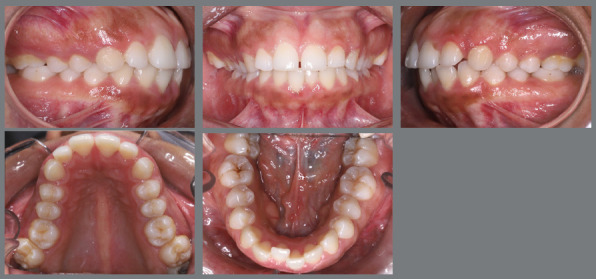
Initial intraoral photographs.

The patient, aged 19, presented with a Class I malocclusion and bimaxillary protrusion, as well as a buccal crossbite of the right second molars (Fig. 28). The mandibular right second molar was extruded and lingually inclined. Before including the second molar in the full arch alignment, creativity was used to correct its position.

**Figure 29:**
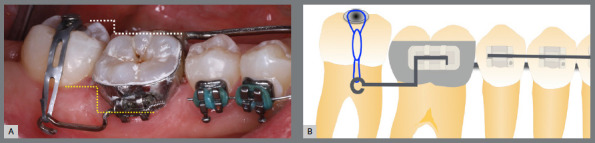
Close-up photograph **(A)** and schematic illustration **(B)** of the mechanics applied.

**Figure 30:**
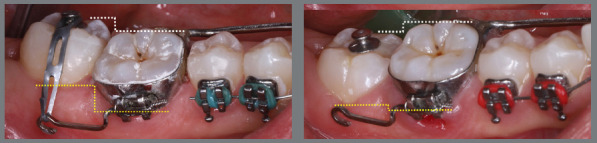
Comparative photographs of the outcome of the mechanics applied.

A rigid (0.9 mm) mandibular lingual arch was placed, positioned away from the incisors, and anchored to the first molars. A bracket was welded to the first molar band, and a 0.021 x 0.025-in segmental wire was fabricated with a distal extension and a cervical hook centered on the second molar’s crown. A button was bonded to the occlusal surface of the second molar, and an elastic chain was applied from the button to the hook, generating intrusive and buccal forces (Figs. 29 and 30).

**Figure 31:**
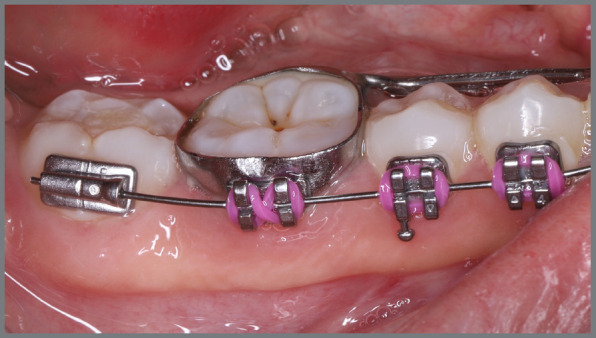
Close-up intraoral photograph of tube bonding on the second molar and alignment and leveling stage.

After three months, the molar’s position was corrected, the button was removed, and a tube was bonded to integrate it into the conventional alignment and leveling sequence (Fig. 31).

### CLINICAL SITUATION 6

#### Disimpaction of a mandibular second molar using a lingual holding arch

**Figure 32:**
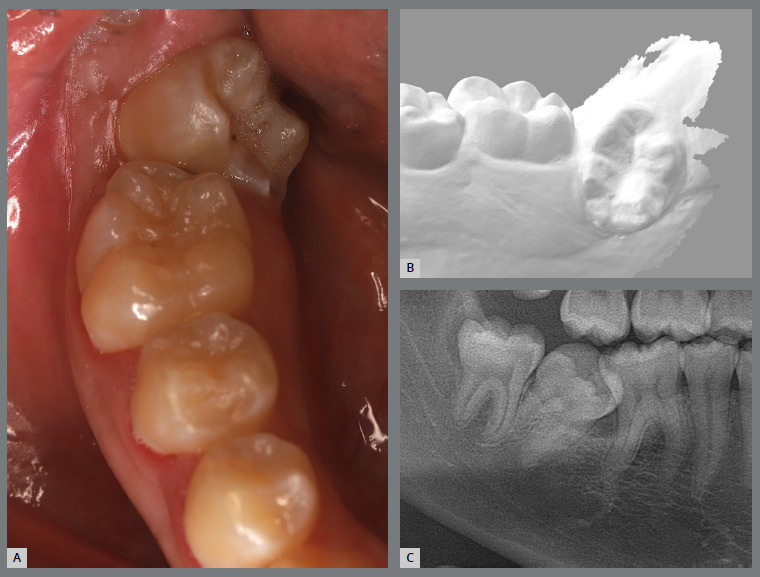
Images of impaction of the mandibular right second molar: **A)**close-up intraoral photograph; **B)** lingual view of the digital scan; and **C)**panoramic radiograph close-up.

The patient, aged 17, presented with severe lingual inclination, mesial angulation, rotation, and mild impaction of the mandibular right second molar against the first molar (Fig. 32). 

**Figure 33:**
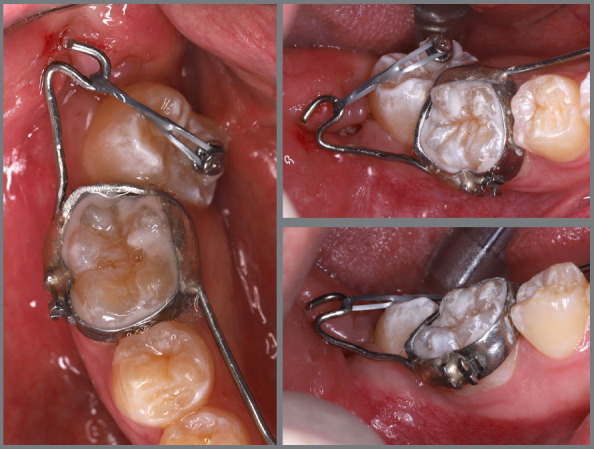
Various views of the mechanics adopted for second molar disimpaction.

A rigid (0.9 mm) lingual arch was installed and anchored to the first molars, with a distal wire extension with a hook soldered to the molar band. After extraction of the third molar, a button was bonded to the occlusal surface of the second molar and, with the use of an elastic chain, distal and buccal forces were generated (Fig. 33). 

**Figure 34:**
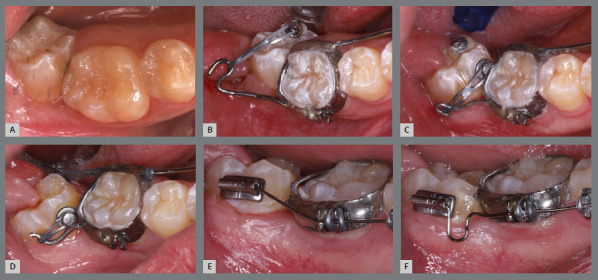
Close-up photographs illustrating the main stages of clinical case progression: **A)** initial; **B)** initiation of mechanics; **C)** change in force line of action with bonding of a new button; **D)** progress of molar disimpaction; **E)** tube bonding on the molar and initiation of alignment and leveling; and **F)** completion of alignment and leveling phase.

After two months, the molar was distalized, but without rotation correction. A new button was bonded to the buccal surface (on the mesial aspect), an adjustment was made to the size of the hook, and a new activation with an elastic chain was performed. After two months, the molar was uprighted, but the rotation had not been fully corrected. The occlusal button was removed, a new button was bonded to the lingual surface, and a new activation was performed using elastic chains on both buttons, creating an orthodontic force couple to correct the rotation. After three months, the second molar was aligned, but not fully leveled. At that moment, a tube was bonded, and the tooth was aligned with flexible wires. After two months, the second molar was fully corrected (Fig. 34).

### CLINICAL SITUATION 7

#### Disimpaction of a mandibular second molar using a lingual holding arch

**Figure 35:**
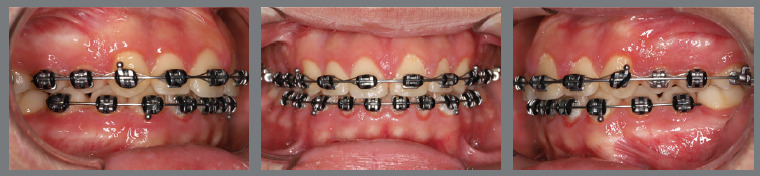
Initial intraoral photographs.

**Figure 36:**
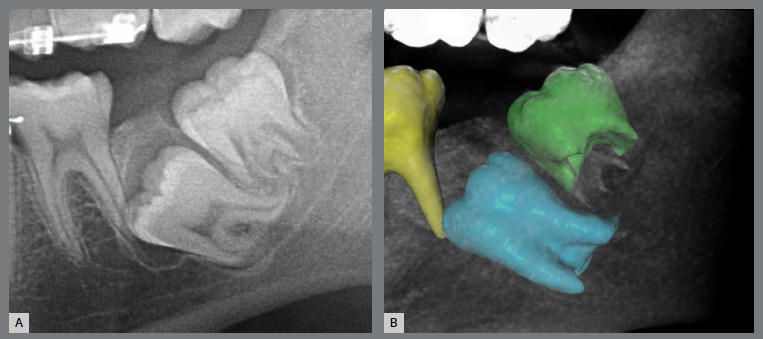
Images of impaction of the mandibular left second molar: **A)**panoramic radiograph close-up; and **B)** cone-beam computed tomography close-up.

The patient, aged 14, was undergoing orthodontic treatment with another professional and came for a consultation. The patient had Class I malocclusion with severe impaction of the left mandibular second molar (Fig. 35). The tooth was mesial and buccally tipped, and its crown was close to the root of the first molar. In addition, the third molar was also mesially tipped with the crown above the second molar (Fig. 36). 

According to the patient´s mother, the treatment plan previously proposed was the extraction of the second and third molars. And it was for this reason that she sought a consultation, to get a second opinion. 

After dialogue with the family, the proposed treatment plan was the removal of the previously installed appliance, extraction of the third molar and traction of the second molar. 

**Figure 37:**
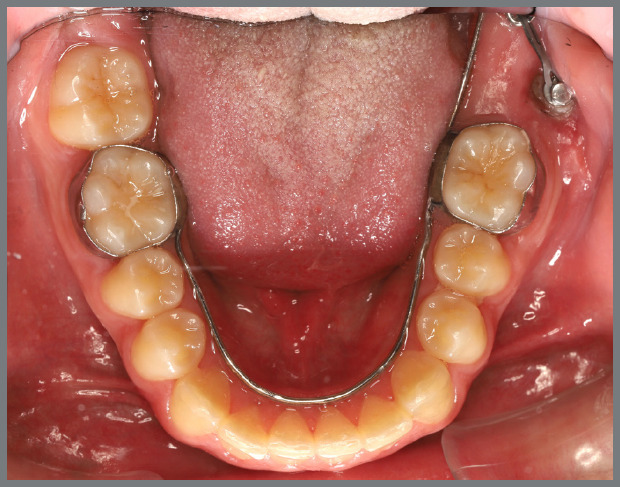
Mandibular occlusal photograph of the mechanics applied.

**Figure 38:**
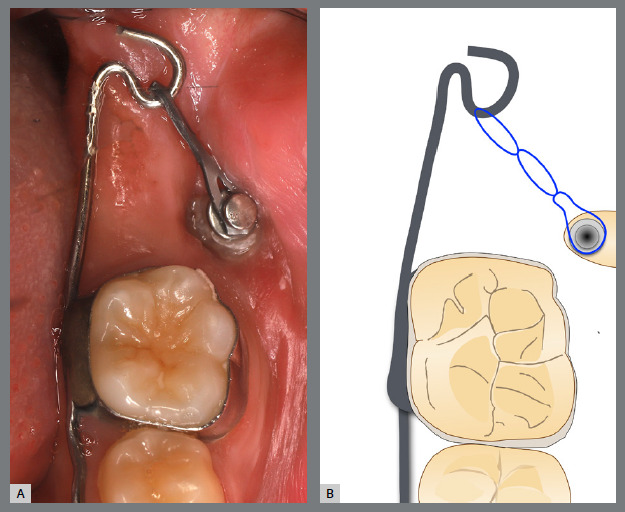
Close-up photograph **(A)** and schematic illustration **(B)** of the mechanics applied.

**Figure 39:**
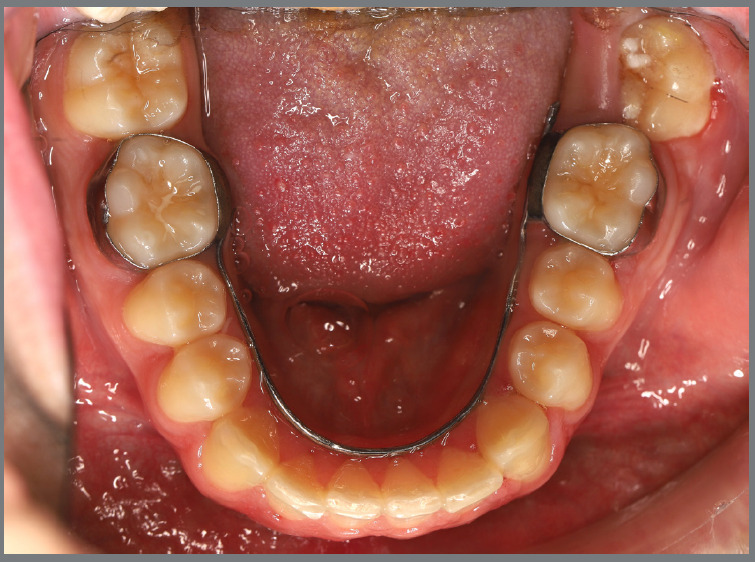
Mandibular occlusal photograph of the outcome of the mechanics applied.

**Figure 40:**
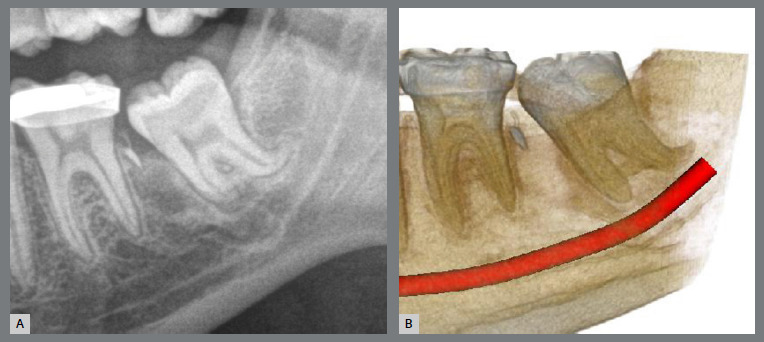
Close-up images of the outcome of molar traction: **A)** panoramic radiograph close-up; and **B)** cone-beam computed tomography close-up.

Similar to the previous case, a rigid lingual arch (0.9mm) was installed away from the incisors and anchored to the first molars with a distal wire extension with a hook. In the same surgical procedure for the third molar extraction, a button was bonded to the buccal surface (on the mesial surface) of the second molar and, with an elastic chain, from the button to the hook, a force was generated in a lingual and distal direction (Figs. 37 and 38). After four months, the molar was disimpacted, but the clinical crown was buccally tipped (Figs. 39 and 40). 

**Figure 41:**
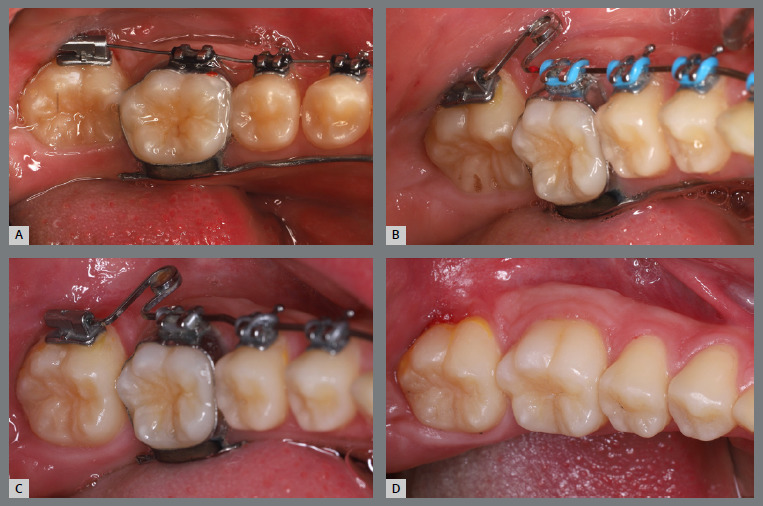
Close-up intraoral photographs illustrating the main stages of clinical case progression after molar traction: **A)** Tube bonding and beginning of alignment; **B)** beginning of torque correction mechanics with loop and rectangular archwire; **C)** completion of torque correction and beginning of finishing; and **D)** post-treatment result.

At this point, the orthodontic appliance was installed, and a tube was bonded to the second molar. Flexible archwires were used to perform molar alignment and leveling. After two months, the second molar was well-positioned, but with a significant change in torque. To correct the torque, a rectangular 0.018 x 0.025-in steel archwire with a vertical loop was installed. After five months, both the position and the torque had been fully corrected, demonstrating the efficacy of the clinical procedures performed (Fig. 41).

### CLINICAL SITUATION 8

#### Optimization of mandibular incisor alignment using lingual buttons

Orthodontic forces are usually applied to the buccal surfaces in both bracket and aligner therapy. When incorporating creativity, the lingual surface can be used as anchorage levers for orthodontic mechanics.

The patient presented with a Class I malocclusion and moderate anterior mandibular crowding. Her chief complaint involved improvement in smile esthetics and the correction of lower crowding. 

**Figure 42:**
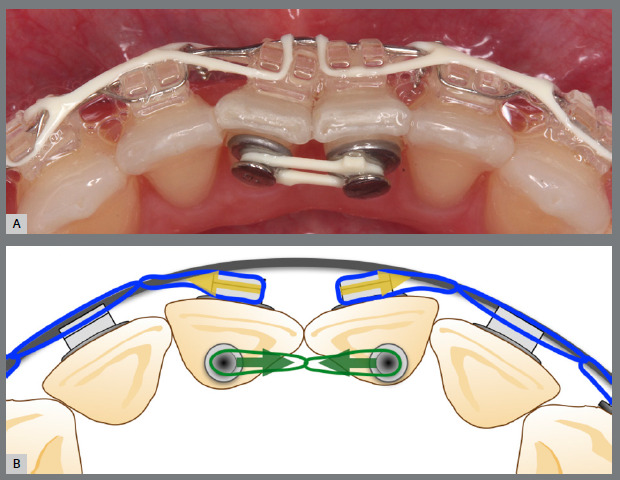
Close-up photograph **(A)** and schematic illustration **(B)** of the mechanics employed.

**Figure 43:**
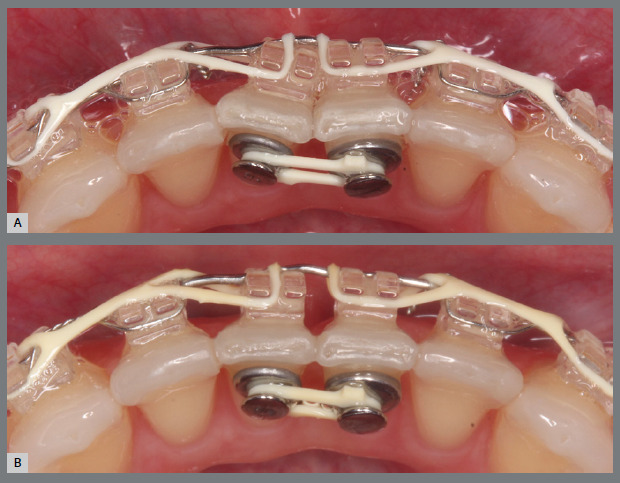
Comparative photographs of the outcome of the mechanics employed.

During the final stage of incisor alignment, central incisors remained malpositioned. By using creativity, we used two orthodontic force couples (Fig. 42). Lingual buttons were bonded to the central incisors, and elastic chains were applied from both buccal and lingual surfaces to create force couples, achieving full correction within 30 days (Fig. 43).

### CLINICAL SITUATION 9

#### Optimization of canine retraction using lingual distalizing force

Following the same concept as the previous case, lingual forces can be advantageous for canine retraction after first premolar extraction.

**Figure 44:**
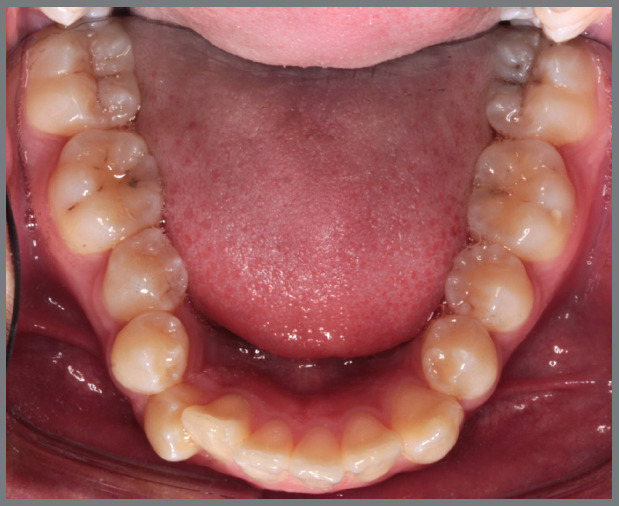
Initial mandibular occlusal intraoral photograph.

The patient presented with a Class I malocclusion, moderate crowding in both arches, and an unfavorable position of the mandibular right canine (Fig. 44). 

**Figure 45:**
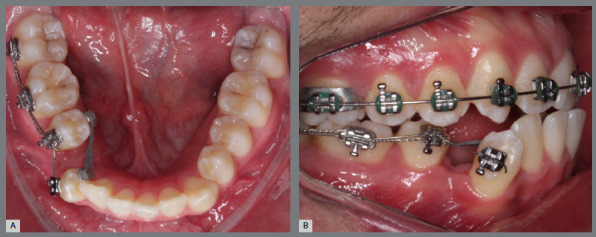
Photographs of the mechanics employed: **A)** mandibular occlusal view; and **B)**right lateral view.

**Figure 46:**
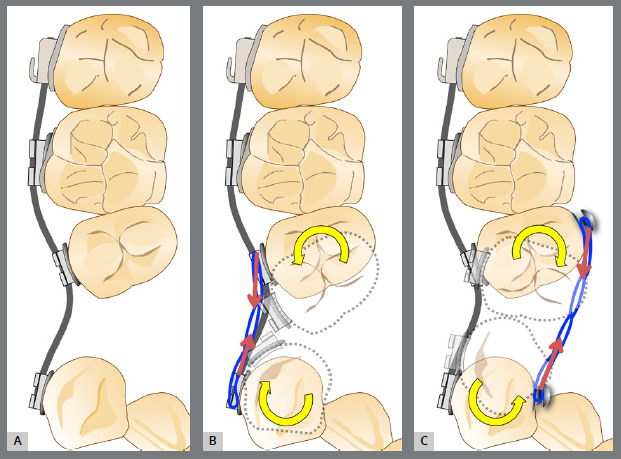
Schematic illustrations of the mechanics employed: **A)** initial condition; **B)** illustration of possible outcome if forces were applied buccally, demonstrating undesirable results; and **C)** illustration of the mechanics employed, demonstrating positive outcomes.

**Figure 47:**
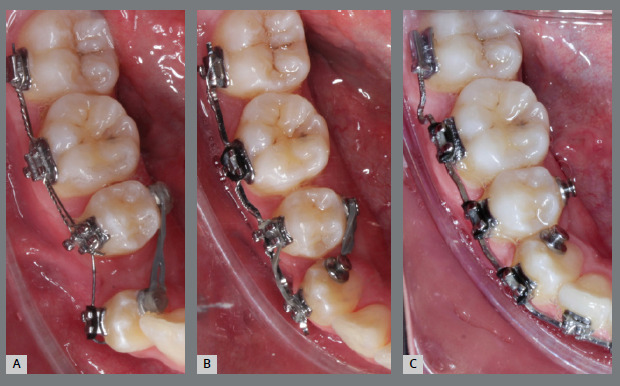
Comparative photographs of the outcome of the mechanics employed: **A)** initial condition; **B)** result after three months; and **C)** clinical situation after space closure.

The mandibular fixed appliance was partially bonded, excluding the incisors. A 0.016-in NiTi wire segment was installed from the second molar to the canine, and the molars and premolars were tied together with steel ligature (Fig. 45). A button was bonded to the lingual surface of the canine and, to optimize retraction with rotation correction, we used a lingual line of force (Fig. 46). As a result, the momentum created by the retraction force would be favorable to the retraction with rotation correction. After three months, the mechanics proved to be quite favorable, and the arch was aligned and leveled, and the spaces were closed (Fig. 47).

### CLINICAL SITUATION 10

#### Correction of bilateral posterior crossbite using a Hyrax in the mandibular arch

In this final case, creativity was applied to correct a bilateral posterior crossbite in an adult patient. 

**Figure 48:**
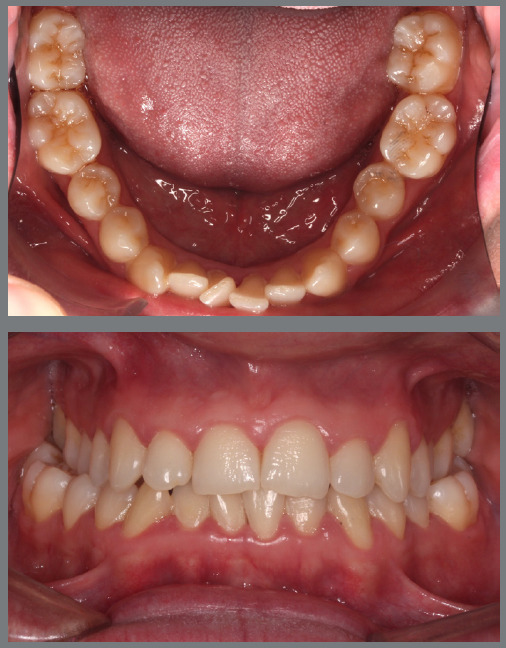
Initial intraoral photographs.

The patient presented with a Class I malocclusion, severe mandibular crowding, and buccally tipped mandibular first molars (Fig. 48).

**Figure 49:**
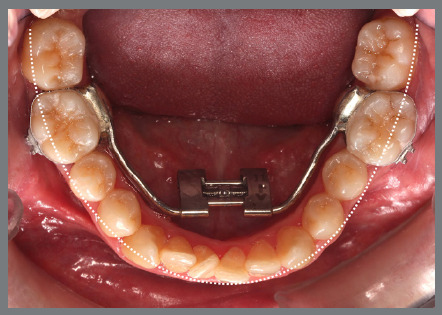
Mandibular occlusal photograph of the mechanics applied.

**Figure 50:**
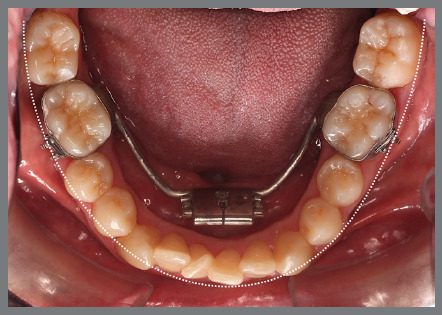
Mandibular occlusal photograph of the outcome of the mechanics applied.

**Figure 51:**
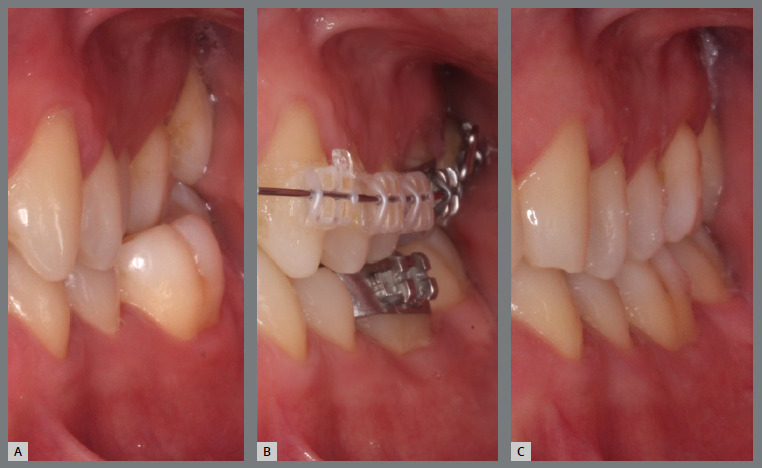
Close-up intraoral photographs illustrating the main stages of clinical case progression: **A)** initial condition; **B)** result after mechanics with Hyrax appliance and initiation of bracket therapy; and **C)** post-treatment result.

A mandibular Hyrax expander was fabricated with the screw pre-opened before soldering to the bands, allowing activation to contract rather than expand the arch (Fig. 49). The patient was instructed to activate the appliance by one-quarter turn three times per week. After four months, the mandibular molars were moved into proper alignment with the arch (Fig. 50). Figure 51 illustrates a close-up view of the frontal left side of the most important phases of the treatment.

## DISCUSSION

Creativity in Orthodontics is an essential clinical skill, particularly considering the increasing complexity of cases and the heightened aesthetic demands of contemporary patients. Beyond the mere application of pre-established protocols, creativity enables the orthodontist to think critically, create individualized solutions, and safely and effectively incorporate technological innovations.^4^ ^,^ ^5^

In clinical contexts, for example, creativity is essential for modifying traditional orthodontic mechanics. Among the cases presented, creativity expanded the applicability of the lingual holding arch for multiple clinical purposes. Of the ten clinical situations described, in five of them the lingual arch was adapted for specific objectives, such as the correction of premolar and canine rotations, as well as the intrusion and/or disimpaction of mandibular second molars. For the disimpaction and/or intrusion of lower molars, mini-implants^10^ ^-^ ^12^ have also been used with satisfactory clinical efficacy in recent years; however, such approaches are more invasive, compared to lingual arch mechanics.

Similarly, the correction of rotations in the maxillary arch can also benefit from creative approaches, such as the use of palatal bars and Nance appliances, as demonstrated in two of the clinical situations. Another creative use of the palatal bar was published for the disimpaction of impacted permanent first molars.^5^

Other reported cases involved the application of orthodontic forces from the lingual side, thereby modifying the conventional *modus operandi* of orthodontic mechanics. In these examples, the decision to apply forces on the lingual surface (with orthodontic buttons) generated beneficial lines of force for the desired movement. Such technique modifications demand refined clinical reasoning and innovative thinking. In clinical situations 8 and 9, for instance, the use of lingual force application produced rapid clinical outcomes, in which the side effects of the forces were advantageous to treatment.

In clinical situation 10, creativity completely altered the approach to treating a posterior crossbite in an adult patient. In this example, unlike the conventional methods described in the literature,^13^ including mini-implant assisted maxillary expansion,^14^ the treatment consisted of mandibular molar contraction, achieved through the modification of a Hyrax appliance.

It is important to emphasize that creativity must be employed responsibly, grounded in the scientific literature and ethical principles. The risk of uncontrolled experimentalism is always present when extrapolating techniques without adequate scientific support. Therefore, creative innovation must always be accompanied by sound clinical reasoning and scientific validation.

In summary, creativity is a skill that should be cultivated throughout the orthodontist’s professional career.^4^ ^,^ ^5^ ^,^ ^15^ It is essential for addressing complex clinical challenges, keeping pace with technological advancements, meeting patients’ esthetic expectations, and innovating responsibly in clinical practice. Contemporary Orthodontics is, therefore, both an exact science and an art; and it is at the intersection of knowledge and creativity that its true transformative potential lies.

## CONCLUSION

Creativity is an essential component of orthodontics. Its integration into clinical practice significantly contributes to individualized treatment, the resolution of therapeutic challenges, and the safe incorporation of new technologies. Encouraging creativity from the early stages of academic training is crucial to preparing professionals capable of innovating responsibly.

## Data Availability

All data generated or analyzed during this study are included in this published article.
